# VIP: an integrated pipeline for metagenomics of virus identification and discovery

**DOI:** 10.1038/srep23774

**Published:** 2016-03-30

**Authors:** Yang Li, Hao Wang, Kai Nie, Chen Zhang, Yi Zhang, Ji Wang, Peihua Niu, Xuejun Ma

**Affiliations:** 1 Key Laboratory of Medical Virology, Ministry of Health; National Institute for Viral Disease Control and Prevention, Chinese Center for Disease Control and Prevention, Beijing, 102206, China; 2 National Engineering Research Center for Beijing Biochip Technology, Beijing 102206, China; 3 Department of Infectious Diseases, Institute of Biomedicine, Sahlgrenska Academy, University of Gothenburg, Gothenburg, 41345, Sweden

## Abstract

Identification and discovery of viruses using next-generation sequencing technology is a fast-developing area with potential wide application in clinical diagnostics, public health monitoring and novel virus discovery. However, tremendous sequence data from NGS study has posed great challenge both in accuracy and velocity for application of NGS study. Here we describe VIP (“Virus Identification Pipeline”), a one-touch computational pipeline for virus identification and discovery from metagenomic NGS data. VIP performs the following steps to achieve its goal: (i) map and filter out background-related reads, (ii) extensive classification of reads on the basis of nucleotide and remote amino acid homology, (iii) multiple k-mer based *de novo* assembly and phylogenetic analysis to provide evolutionary insight. We validated the feasibility and veracity of this pipeline with sequencing results of various types of clinical samples and public datasets. VIP has also contributed to timely virus diagnosis (~10 min) in acutely ill patients, demonstrating its potential in the performance of unbiased NGS-based clinical studies with demand of short turnaround time. VIP is released under GPLv3 and is available for free download at: https://github.com/keylabivdc/VIP.

The world contains a high diversity of human viral pathogens. There are approximately 300 recognized viral pathogen species, and additional species continue to be discovered. The identification of viral pathogens has a tremendous impact on infectious diseases, virology and public health. Nearly all of the outbreaks of public health issues over the last decade have been caused by viruses, including Severe Acute Respiratory Syndrome (SARS) coronavirus^1^, 2009 pandemic influenza H1N1^2^, H7N9 avian influenza viruses^3^ and the recently described Ebola virus in West Africa^4^. Traditional diagnostic methods for viruses, such as cell culture, serodiagnosis, or nucleic acid-based testing are narrow in scope and require a priori knowledge of the potential infectious agents^5^,^6^. Accurate diagnosis and timely treatment for the infection dramatically reduced the risk of continued transmission and mortality in hospitalized patients^7^. Wild interest in comprehensive detection of these newly emerging and re-emerging viruses from clinical samples highlight the need for rapid, broad-spectrum diagnostic assays.

Shotgun metagenomic sequencing of clinical samples for viral pathogen identification provides a promising alternative solution. Although metagenomics is typically applied to understanding genomic diversity from environment samples, this methodology has also revolutionized virology with comprehensive applications, including viral pathogen identification of infectious disease in clinical laboratories^8^ and virus discovery in acute and chronic illnesses of unknown origin^9^. Many novel viruses have been discovered using popular next generation sequencing (NGS) platforms such as pyrosequencing (454 Roche), semiconductor sequencing (Life Technology) and illumina dye sequencing (Illumina)^10^,^11^,^12^. Achievements obtained by viral metagenomics show significant advantages over traditional methods of identifying a viral pathogen, including no need of sequence information for that pathogen, identifying multiple pathogens in a single assay and eliminating the need for time-consuming culturing or antibody laboratory tests.

A key feature of latest NGS platforms is their speed. It takes minimum turnaround times about 8 hours for sequencing^13^. Thus, it is critical that subsequent computational handling of the large amount of sequence data generated in viral metagenome sequencing must be performed within a timeframe suitable for actionable responses. Most commercial NGS services, however, offer basic bioinformatics support such as *de novo* sequence assembly or mapping to reference genomes, but will not process further to the specifics of virus identification and discovery. There are many bioinformatics tools developed specifically for virus detection from NGS data. In general, the strategies in these pipelines are computational subtraction to pathogen detection. Reads corresponding to the host (e.g., human) are first removed, followed by alignment to reference databases (DB) that contain sequences from candidate pathogens^14^,^15^,^16^,^17^,^18^. The most common for virus identifying are local alignments with reference DB, such as the Basic Local Alignment Search Tool (BLAST) algorithm^19^. Analysis pipelines that use faster algorithms (e.g., Bowtie or Bowtie2) for host computational subtraction, such as VirFinder^15^ and VirusFinder^16^ rely on traditional BLAST approaches for final pathogen determination. BLAST is generally used in these tools for classification of viral reads at the nucleotide level (BLASTn), followed by less stringency protein alignments using a translated amino acid alignment (BLASTx) for identification of novel viruses with divergent genomes. However, BLAST is too slow for massive data from NGS. For example, end-to-end processing times, even on multicore computational servers, can take several days to weeks^14^. Another issue is related to *de novo* assembly. The majority of pipelines utilized to assemble metagenomics data were originally developed to assemble single genomes, However, single-genome assemblers were not aimed to assemble multiple genomes from metagenomics data which were with nonuniform sequence coverages^20^. Problems in the assembly results may include chimeric contigs (reads artificially combined during assembly) which are not easy to be recognized. Additional limitations of these available bioinformatics software for viral pathogen identification include high hardware requirements (multicore servers), the need for bioinformatics expertise, and the lack of validated and clear results to permit confident identification of viruses from metagenomics NGS data. Biologists often have to rely on professional bioinformaticians to process NGS data, posing a bottleneck in data analysis.

Here we present VIP (Virus Identification Pipeline), a one-touch bioinformatic pipeline for virus identification with fairly self-explanatory results in Hypertext Markup Language (HTML). VIP performs extensive classification of reads against DB collected by Virus Pathogen Resource (ViPR)^21^ and Influenza Research Database (IRD)^22^ nucleotide DB in fast mode and against the virus sequences with NCBI Refseq (http://www.ncbi.nlm.nih.gov/refseq/) and their neighbor genomes in sense mode (Fig. 1). Novel viruses are firstly identified in sense mode by amino acid alignment to NCBI virus Refseq amio acid DB and followed by phylogenetic characterization. Notably, VIP generates results in a clinically actionable timeframe of minutes to hours with lower hardware requirements by leveraging two alignment tools, Bowtie2^23^ and RAPSearch^24^, which have computational times that are orders of magnitude faster than BLAST packages. Here we evaluate the performance of VIP for viruses characterization using various NGS datasets public available generated by cross-platforms (454, Ion Torrent and Illumina). These data sets encompass a variety of clinical infections, detected pathogens, sample types, and depths of coverage. We also demonstrate the use of the pipeline for detection of re-emerging dengue virus 1 (DENV-1) from a case with a febrile status in Guangzhou.

## Results

### Accuracy of the classification strategy using in-silicon data

**Figure 2:**
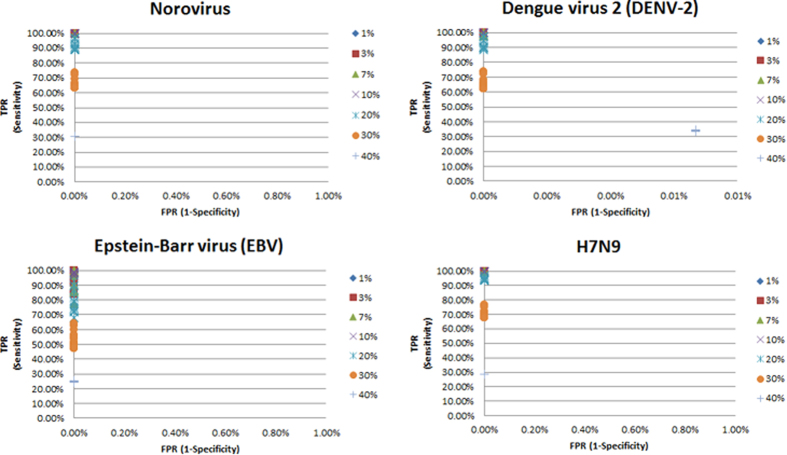
The classification method in VIP was evaluated from in-silicon query data set generated from 4 viruses including Dengue Virus 2 (DENV-2), Epstein-Barr virus (EBV), Norovirus and H7N9. ROC curves were generated to evaluate the ability to correctly detect in silico-generated NGS reads at different mutation rate. Sensitivity or the true positive rate (TPR) (y-axis) is plotted against 1-specificity or the false positive rate (FPR) (x-axis).

In order to evaluate the accuracy of the two-steps classification strategy (sense mode), a query data set of 125 base pair (bp) reads was generated in-silicon. The viral reads were generated at different mutation rate ranging from 1% up to 40% from four known representative viruses which were Dengue Virus 2 (DENV-2, a single positive-standed RNA), Epstein-Barr virus (EBV, a double stranded DNA), Norovirus (a genetically diverse group of single-stranded RNA) and H7N9 (segmented negative-sense RNA) using wgsim (https://github.com/lh3/wgsim). Receiver operating characteristic (ROC) curves^25^ were generated to assess the sensitivity and specificity of the classification methods at different mutation rate in classifying viral reads. (Figure 2 and see Supplementary Information) Nearly all the results shared the superior 100% specificity. The specificity was slightly reduced (99.83%) when classifying the DENV-2 viral reads at 40% mutation rate. The sensitivity was reduced while the mutation rate was increased. Accurate virus identification, where sensitivity >90%, was still achieved for with mutation rate at 20% using optimal parameters. Nevertheless, the overall poor performance in classifying the viral reads at high mutation rate (>20%) required further investigations.

### Performance comparison between assembly software packages and assembly strategy in VIP

In this study, we also compared the *de novo* assembly performance for VIP, IDBA_UD^26^ and recently described Ensemble Assembler^27^ via using public datasets, in-house datasets and in-silicon dataset at mutation rate 3% with viruses which were DENV-2, EBV, Norovirus and H7N9. In-house Perl scripts were developed to calculate assembly metrics. The different assembly statistics calculated for each assembly results in order to assess the performance of each assembly are shown in Table 1. This table describes the overall length and genome coverage estimators per assembly including the N50, percent of genome coverage of largest contig (%largest_contig_coverage) and percent of all contigs (%contig_coverage).

In general, VIP prominently improved the N50 and obtained overall better outcomes (Table 1). For example, in in-house dataset 1, N50 acquired by VIP is 6058, while Ensemble Assembler and IDBA_UD were 207 and 358 respectively. The same trends were also found for percent of genome coverage of largest contig and percent of all contigs coverage. Two datasets SRR453448 and SRR1106553 with IDBA_UD carried out slightly better results than VIP. The simulation data set was to evaluate the assembly performance and VIP demonstrated to build the longest contig, along with highest percent of contig coverage and largest contig coverage for all four kinds of viruses.

### Accurate identification of viruses from various NGS datasets

To evaluate VIP, we first tested the ability of VIP to detect the presence of viruses in various samples generated from different sequencing platforms. Table 2 lists the NGS datasets available at Sequence Read Archive (SRA; http://www.ncbi.nlm.nih.gov/sra/) for our benchmark experiment. These samples were infected with viruses of diverse types and all of results were confirmed by independent experiments.

As indicated in Table 1, VIP identified the same virus type or subtype in all the test samples. For example, the viruses from data source SRR1106123 was isolated from serum, Hepatitis C virus and Hepatitis G virus were found with VIP pipeline, and the genome coverage were both 100% for the two identified viruses, which was confirmed by independent experiments. Furthermore, the VIP has the power to discover more undetected viruses from the dataset. In data source SRR1170820 from Ion Torrent PGM platform, Bovinecircovirus (BoCV) was reported by traditional test, while with VIP in addition to the Bovinecircovirus, the Bovine Viral Diarrhea Virus (BVDV) was also identified, and the genome coverage were 99.90% and 86.13% respectively. We also evaluated VIP using additional 3 in-house samples from different sequencing platforms, including 2 mixed samples (sequenced by Hiseq 2000 and 454 respectively). These 3 in-house samples were isolated from stool, serum and swab respectively. For the mixed swab samples, we found Respiratory Syncytial Virus (RSV) and Human coronavirus 229E using our method, and the genome coverage were 98.41% and 17.00% respectively, it is worth noting that the number of reads of Human coronavirus 229E was only 262, which was merely 0.01 percent of its total reads (5,147,814 reads), the presence of Human coronavirus 229E was then confirmed by nested PCR experiment. VIP was able to correctly identify the virus types for all of these samples and hence demonstrated its robustness in virus detection.

We selected 3 sets of data from SRA to evaluate the sensitivity and specificity of VIP to identify candidate virus in clinical metagenomics datasets. Note that the 3 datasets results have been confirmed by related experiments and the genbank accession number of each reference sequence was introduced. Sensitivity and specificity were used to evaluate the ability of classification algorithm. Sensitivity, or true positive rate (TPR), was calculated as TPR = TP/(TP + FN). Specificity, or true negative rate (TNR), was calculated as SPC = TN/(TN + FP), in which TP and TN represent true positives and true negatives, and FN and FP represent false negatives and false positives. The gold standard criterion for a correct viral classification was BLASTn alignment against the target viral genome at an E-value cutoff of 10^−8^ 14. According to the results, the sensitivity were ranged from 97.03% to 99.83%, and the specificity were all above the 99.89%. (see Supplementary Table S1).

### Speed of VIP and feasibility for real-time clinical analysis

**Figure 3:**
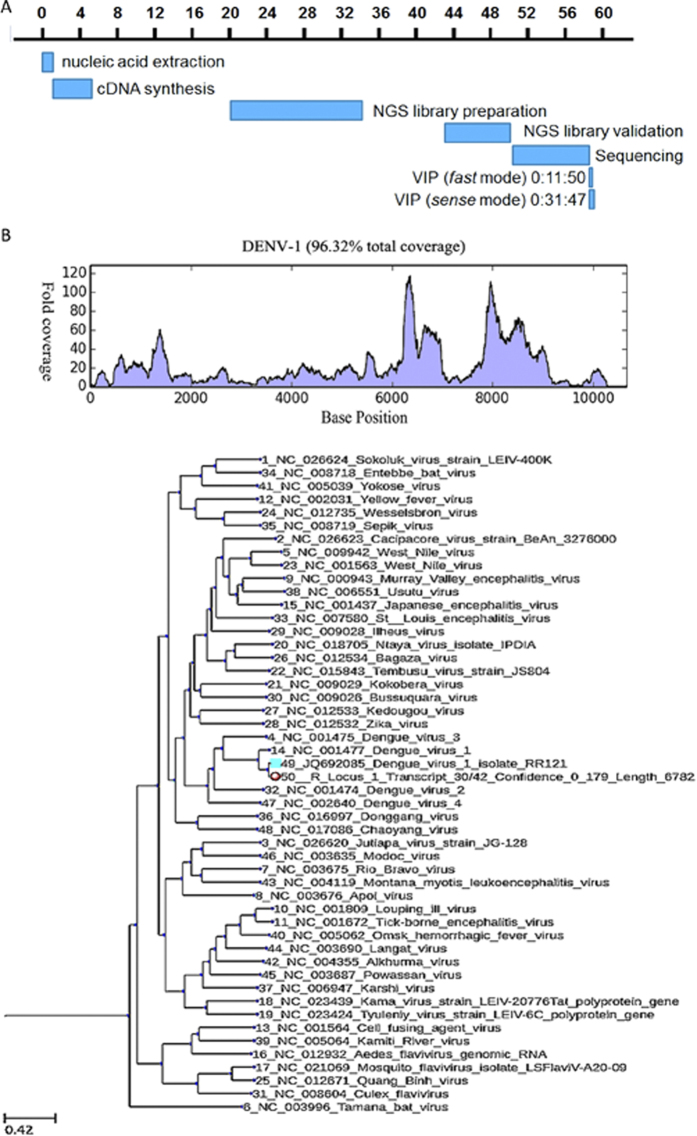
Speed of VIP and feasibility for real-time clinical analysis. (**A**) a clinical sample from Guangzhou with a febrile status was analyzed using NGS, resulting in VIP detection in a 60-hr timeframe from sample to answer. (**B**) VIP results suggested primary DENV-1 infection. Percentage coverage of DENV-1 achieved using VIP was 96.32%. Further *de novo* assembly result showed it was clustered into DENV-1 group (red circle). The reference genome was labeled as blue square.

NGS and VIP analyses were applied to analyze a clinical sample from 2014 Guangzhou Dengue outbreak to test the speed and feasibility of VIP. As is shown in Fig. 3, within a 60-hr sample-to-answer turnaround time and ~10 min VIP analysis time (fast model), nearly complete genome (96.32%) of dengue virus 1 (DENV-1) was obtained, and the fold coverage for coding regions was better than that for non-coding regions. The subsequent phylogenetic tree also located this sequence to DENV-1 branch, confirming the percentage coverage result. As no other pathogens were identified with VIP pipeline, subsequent confirmatory real-time PCR assay was carried out to verify the VIP’s result, and the real-time PCR correctly amplified the genome of DENV-1, which supported the primary conclusion of DENV-1 infection. The patient recovered spontaneously without any complications.

## Discussion

The state-of-the-art NGS technology is becoming mature and is increasingly introduced in routine laboratories. The price of NGS is falling dramatically in some cases below the price of traditional identification methods. NGS has emerged as one of the most promising strategies for the detection of pathogens in clinical samples. Viral metagenomics provides comprehensive viral pathogen identification in a single assay. However, bioinformatics analysis for viral metagenomics has been the bottleneck when deploying NGS for virus identification due to the absence of a designated bioinformatician in most laboratories. Our goal is to establish a one-touch computational pipeline in routine laboratories for virus identification from metagenomics NGS data generated from complex clinical samples.

Latest described SURPI was an ultrafast computational tool for pathogen identification. Both SURPI and VIP shared generally the same strategy “subtraction to identification”. SURPI searched against the entire both nucleotide and amino acid collection from NCBI (nt/nr) for comprehensive identification of pathogen. Coverage map, a very attractive feature provided in SURPI, was also carried out in VIP with strategy as previously described. The major difference was the way to select the close reference. SURPI would map to all nucleotide reference sequences corresponding to that genus picked out during alignment. VIP will choose a close reference based on abundance of reference genome due to the proposed hypothesis that the genome coverage percentage was alongside with the sequencing depth. The approaches for assembly share little similarity. VIP implemented the “classification to assembly” while SURPI used the entire data set to perform assembly. Results suggested higher N50 were achieved by VIP. (Data not shown) Compare to other pathogens, like bacteria, fungi and parasite, most viral pathogens are RNA viruses which suggested the potentially high mutation rate or divergent genomes. It could be impossible to find a suitable reference genome during alignment procedure in some cases which undermined the coverage map and underscored the phylogenetic analysis. The construction of a phylogenetic tree allows us to visualize the underlying genealogy between the candidate divergent virus and existing reference sequences. The overall effective *de novo* assemblies benefitted from the classification method in VIP generated longer and accurate contig to help construct the phylogenetic tree. Nevertheless, SURPI was proved to provide more comprehensive information for pathogen identification by searching against nt/nr while VIP focused on viruses. (see Supplementary Table S3 and S4).

To our knowledge, VIP is the first pipeline to provide both genome coverage map and phylogenetic analysis for virus identification from metagenomics data and has been rigorously tested across multiple clinical sample types representing a variety of infectious diseases. VIP can process the NGS datasets with different NGS formats of different read lengths which suggested the universality of VIP. Notably, VIP can efficiently handle NGS data generated from animals and complex metagenomics samples such as stool and respiratory secretions, which are exposed to the environment and contain a large proportion of non-host sequences (Table 1). The two-step classification approach achieved high sensitivity and specificity where virus mutation rate were less than 20%. (Figure 2) Finally, VIP performs classification and multiple k-mer *de novo* assembly strategy to generate longer contigs for identification of divergent viral sequences. Results of VIP can be easily accessible through web browsers providing intuitional information including the summary table, genome coverage map and phylogenetic tree figure. (see Supplementary Figure S1).

Practical application indicates that VIP can be performed within a timeframe suitable for actionable responses in clinical settings. In 2014, there was a severe Dengue outbreak in Guangzhou, Guangdong province, causing more than 40 thousand laboratory confirmed cases^28^. We used IonTorrent PGM platform to sequence one unknown sample from this outbreak, the new developed VIP was then utilized to analyze the sequencing data, and we successfully obtained the nearly complete DENV-1 genome and the result was confirmed by traditional detection method, which indicates the potential applications in the rapid response to emergency outbreak. (Figure 3A) In addition, we also applied NGS and VIP to analyze the first imported MERS-CoV (Middle East Respiratory Syndrome Coronavirus) case in China. Within 36 hr, the full genome of MERS-CoV was obtained and confirmed by reverse transcription-PCR^29^.

VIP also makes significant contributions for virus discovery. We recently analyzed cell culture supernatants and found considerable amount of reads were classified under the orbivirus genus. The orbivirus reads, however, shared less than 50% identity with several specific segments. The phylogenetic report showed the one of the contigs was included in the branch of Tibet orbivirus. The *de novo* assembly results from VIP provided insights to design virus-specific primers. Following specific PCR to confirm the assembly result and fill the gaps, nearly full genome of this virus were recovered (manuscript accepted). Another powerful application by NGS for virus identification is a single step of high-throughput parallel sequencing of small RNAs^30^. The datasets available online^30^ were subjected to VIP and the results were not only corresponding to the literature report also expanding the candidate virus types. (see Supplementary Table S2).

Taken together, our results demonstrate that the proposed VIP combined with NGS has the advantages of simplicity, rapidity, universality and feasibility in the applications of virus detection and discovery. In addition to maintaining the software, we are currently upgrading VIP including development of a user-friendly graphical interface (GUI) based on stand-alone web interface. Therefore, VIP shows the great potential to be standardized and readily and freely accessible to a wider audience of scientists in routine laboratories. Bioinformatics analysis is no longer the weak link when applying NGS as a diagnostic tool for infectious diseases.

## Materials and Methods

The VIP is comprised of a series of Shell, Python, and Perl scripts in Linux and incorporates several open-source tools. VIP has a set of fixed external software and database dependencies and user-defined custom parameters. The pipeline accepts cross-platform results generated from 454, ion torrent and Illumina with a variety of formats such as FastQ, FastA, SAM and BAM alignment formats. Reads are handled by concatenating the files into a single file for streamlined analysis.

### Data import and quality control

Raw NGS short read data can be imported by trans-formatted into FastQ format using PICARD (http://picard.sourceforge.net). VIP will determine the encoding version of the input data. This is necessary to make sure those differences in the way the quality scores were generated from different sequencing platforms are properly taken into consideration during preprocessing. VIP can also accept FastA format raw NGS data. The quality control step, however, will only perform sequence-based strategies, such as the complexity and length as main factors. Generally, the quality control step is comprised of trimming low-quality and adapter reads, removing low-complexity sequences using the DUST algorithm and retaining reads of trimmed length >20 bp using PRINSEQ^31^. In fast mode, Bowtie2 alignments are first performed against the host DB followed by removing the host-related sequences. The remaining reads are subject to ViPR/IRD nucleotide DB. In sense mode, the initial alignment against host DB and bacteria DB is followed by subtraction of reads mapped. Background-subtracted reads are then subject to a virus subset of NCBI nt DB.

### Extensive classification and coverage map

**Figure 4:**
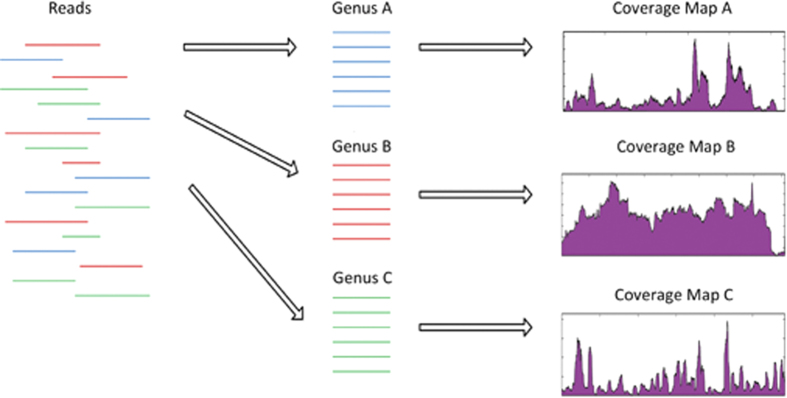
Extensive classification and coverage map. All high quality reads were subject to viral database using nucleotide alignment (Bowtie2) or amino acid alignment (RAPSearch2). Reads would be classified into each viral genus based on alignment results. VIP would choose a close reference based on abundance of reference genome due to the proposed hypothesis that the genome coverage percentage was alongside with the genome sequencing depth. Candidate reads would map against to the selected reference with optimal BLASTn at reward/penalty score (1/−1), followed by coverage map generation.

Previous reports suggested that viruses have the potential to mutate rapidly or jump between species. Reads from mutation region of these viruses might be unclassified or classified into other species or strains. In order to avoid the misclassifications caused by mutations, we applied a two-steps computational alignment strategy for classification in VIP (Fig. 4). In the first step, all matched reads will be assigned to a specific gene identifier (GI) after nucleotide/amino acid alignment. These reads are therefore taxonomically classified to genus level by lookup of matched GI from the NCBI taxonomy database by SQL (Structured Query Language). i.e According to the GI, the scientific names for each reference records, which are composed of the species, genus and family information, are achieved and appended to the alignment results.

Secondly, reads classified under genus are automatically mapped to the most likely reference genome as follows. Abundance of reference sequences that are selected during nucleotide alignment corresponding to that genus are calculated and sorted. Here we hypothesized the genome coverage percentage was alongside with the sequencing depth for specific reference sequences. In other words the higher abundance of a genome suggested the higher possibility to recover its genome. All the reference sequences with the following key words are kept: (1) complete genomes; (2) complete sequences; or (3) complete cds. Assigned reads are directly mapped to all nucleotide reference sequences selected using optimal BLASTn at reward/penalty score (1/−1). The optimal score strategy is most suitable for sequences with low conserved ratio^32^. For each genus, coverage map(s) for the reference sequence(s) were then generated.

### Phylogenetic analysis and multiple k-mer based *de novo* assembly

**Figure 5:**
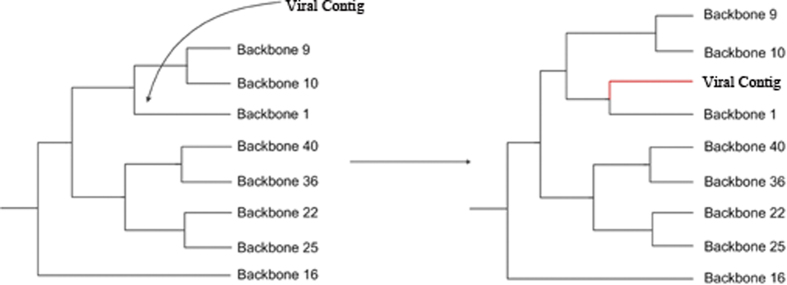
Sequences with Refseq standards under that genus and reference sequence which is used to generate the coverage map are used to generate backbone. Multiple-k method with various k-mer lengths are used for *de novo* assembly with Velvet-Oases. Best contig is then added into the backbone with unweighted pair-group method with arithmetic means (UPGMA) and visualized by Environment for Tree Exploration (ETE).

The construction of a phylogenetic tree allows us to visualize the underlying genealogy between the contiguous sequences and reference sequences. In order to perform a phylogenetic analysis of candidate viruses in a certain viral genus, a backbone with high quality and wide spectrum is indispensable. For a genus, sequences with Refseq standard sunder that genus and the reference sequence which is used to generate the coverage map are selected to carry out multiple sequence alignment to build a backbone using MAFFT^33^ (Fig. 5).

The *de novo* assembly step benefits from the classification method in VIP for significant reduction of complexity of reads. Still *de novo* assemblies from virus samples, especially RNA viruses, into a genome sequence is challenging due to extremely uneven read depth distribution caused by amplification bias in the inevitable reverse transcription and PCR amplification process during library preparations. We present the Multiple-k method in which various k-mer lengths are used for *de novo* assembly with Velvet-Oases^34^,^35^,^36^. In case that sparse reads do not overlap sufficiently to permit *de novo* assembly into longer contiguous sequences, assigned reads and contigs are retained if they are the most appropriate empirical >1.5 × longer than the average length of the candidate reads. Finally the largest contig after *de novo* assembly is added into the backbone to generate phylogenetic tree by unweighted pair-group method with arithmetic means (UPGMA) and visualized by Environment for Tree Exploration^37^.

### Reference database

A 3.8 gigabase (Gb) human nucleotide database (human DB) was constructed from a combination of human genomic DNA, unlocalized DNA (GRCh38/hg38), ribosomal RNA (rRNA, RefSeq), RNA (RefSeq), and mitochondrial DNA (RefSeq) sequences in NCBI as of July of 2015. The viral nucleotide databases in fast mode were constructed from a combination of sequences in ViPR/IRD as of July of 2015. The viral nucleotide DB in sense mode consisted of 87071 entries was constructed by collection of all Refseq viral complete genomes and their neighbor genomes. The neighbor genomes were the complete viral nucleotide sequences which were non-RefSeq recorded from DDBJ, EMBL and GenBank. The viral protein databases were constructed from NCBI Refseq DB. The bacterial DB in sense mode was constructed from the collections of unique genome segments at species level within GOTTCHA^38^ package.

### Hardware

VIP is tested on a common desktop PC with a 3.4 GHz Intel Core i7-4770 and 16 GB of RAM (running Ubuntu 14.04 LTS). Minimum hardware requirements for running VIP include a 4 GB of RAM, PC running Ubuntu 14.04 LTS (preferred). VIP and its external dependencies require about 500 MB of disk space. Reference data requires about 70 GB of disk space. During VIP runtime, up to 10 × the size of the input file may be needed as additional temporary storage.

## Additional Information

**How to cite this article**: Li, Y. *et al.* VIP: an integrated pipeline for metagenomics of virus identification and discovery. *Sci. Rep.* **6**, 23774; doi: 10.1038/srep23774 (2016).

## Supplementary Material

Supplementary Information

## Figures and Tables

**Figure 1:**
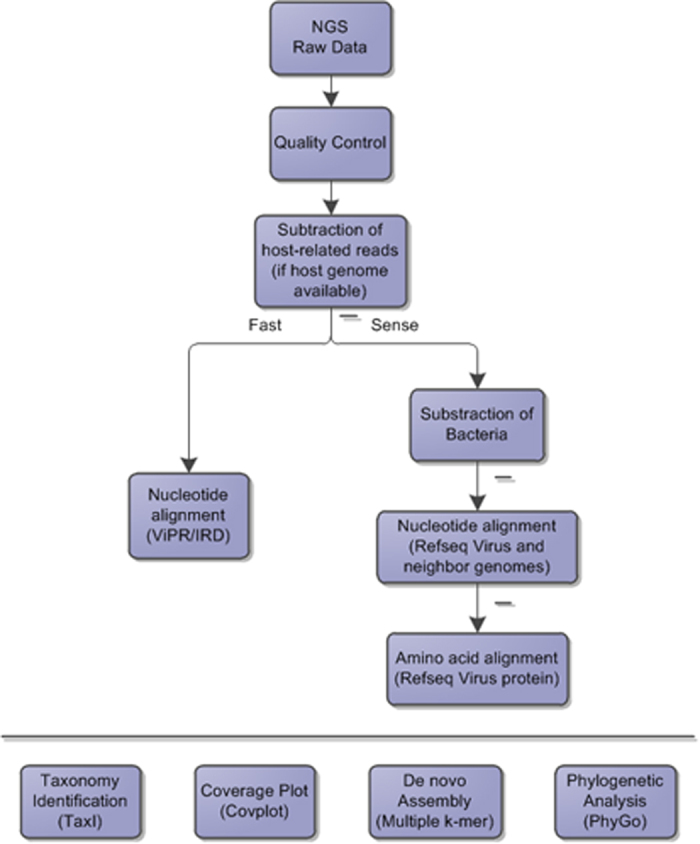
A schematic overview of the VIP pipeline. Raw NGS reads are first preprocessed by removal of adapter, low-quality, and low-complexity sequences, followed by computational subtraction of host-related reads using Bowtie2. In fast mode, viruses are identified by Bowtie2 alignment to ViPR/IRD nucleotide DB. In sense mode, bacteria and related rRNA (ribosomal RNA) reads are removed and the remaining reads are aligned to virus database. Unmatched reads are then aligned to a viral protein database from NCBI Refseq DB using RAPSearch. All matched reads are classified under a genus for *de novo* assembly and phylogenetic analysis. VIP reports include reads distribution, a summary table of classified reads with taxonomic assignments. In addition, results of phylogenetic analysis and genomic coverage map are attached.

**Table 1:** Performance comparison between assembly software packages and assembly strategy in VIP.

Dataset	Virus	N50	VIP		N50	Ensembleassembler		N50	IDBA_UD	
			%contig_coverage	%largest_contig_coverage		%contig_coverage	%largest_contig_coverage		%contig_coverage	%largest_contig_coverage
SRR1170820	BOCV	1184	95.42	11.37(3530)	193	95.94	11.31(3510)	491	96.22	11.89(3691)
	BVDV	528	46.29	7.11(872)		81.99	6.20(761)		36.95	8.86(1087)
SRR453448	DENV-2	1358	98.65	35.78(3837)	167	93.67	30.94(3381)	1572	86.81	39.40(4225)
SRR1106548	HIV	1739	97.02	55.17(5066)	206	88.32	22.89(2102)	356	93.84	46.98(4314)
SRR1106553	H1N1	543	100	59.74(1370)	283	95.68	28.12(645)	1349	67.2	58.78(1348)
In-house-1	DENV-1	6057	95.1	89.53(9612)	207	89.76	9.48(1018)	358	91.36	91.02(9771)
In-house-2	HpeV-3	1690	97.24	45.21(3298)	537	99.34	42.5(3100)	594	94.11	22.42(1636)
	Norovirus GII	2038	93.29	68.57(5182)		92.47	44.96(3398)		92.9	73.74(5573)
	Echovirus-9	669	65.32	12.84(957)		73.12	11.92(889)		56.4	11.25(839)
In-house-4	hPIVs	2056	94.01	21.35(3302)	207	99.71	66.1(10221)	611	99.39	7.45(1152)
	HcoV-HKU1	2022	99.77	51.31(15357)		99.44	29.89(8946)		99.64	31.31(9372)
	HcoV-229E	4372	99.12	29.07(7941)		98.81	28.86(7886)		97.03	16.51(4512)
	RSV	3675	99.29	53.71(8178)		97.83	41.25(6281)		96.55	26.16(3984)
Simulation Data at mutation rate 3%	H7N9	1627^Φ^	N/A	99.73(2291)	520	N/A	99.08(2276)	930	N/A	99.08(2276)
	EBV		N/A	2.27(3938)		N/A	0.81(1401)		N/A	2.06(3566)
	DENV-2		N/A	99.95(10718)		N/A	52.1(5587)		N/A	48.54(5206)
	Norovirus		N/A	99.88(7645)		N/A	40.88(3129)		N/A	35.83(2743)

Abbreviations: BoCV, Bovine enteric calicivirus; BVDV, Bovine viral diarrhea virus; DENV-2, Dengue virus 2; DENV-1, Dengue virus 1; HpeV-3, Human parechovirus 3; hPIVs, Human Parainfluenza Viruses; HIV, Human Immunodeficiency Virus; RSV, respiratory syncyctial virus; EBV, Epstein-Barr virus; HcoV, Human coronavirus; N/A, not available. ^Φ^Every assemble results were compressed into a single file.

**Table 2:** Detection of viruses in various viral metagenomics datasets.

Platform	Data source	Sample type	Independent confirmatory testing results	VIP results	Genome Coverage (%)	Total reads	Number of reads (%)
HiSeq 2000	SRR453448	serum	Dengue virus 2	Dengue virus 2	99.14	1,678,692	64,778(3.86)
HiSeq 2000	SRR453458	serum	Hepatitis A virus	Hepatitis A virus	57.15	446,961	2,315(0.52)
HiSeq 2500	SRR1106123	serum	Hepatitis C virus	Hepatitis C virus	100.00	11,204,134	108,274(0.97)
			Hepatitis G virus	Hepatitis G virus	100.00	11,204,134	751,691(6.71)
MiSeq	SRR1106548	plasma	HIV	HIV	96.61	2,030,011	29,528(1.45)
Genome Analyzer IIx	SRR1106553	nasal swab	H1N1	H1N1	98.25	773,835	12,613(1.63)
Ion Torrent PGM	SRR1170820	nasal swab	BoCV	BoCV	99.90	173,472	9,767(5.63)
				BVDV	86.13	173,472	387(0.22)
Ion Torrent PGM	SRR1170797	buffy coat	BVDV	BVDV	100.00	95,452	33,454(35.05)
Ion Torrent PGM	In-house-1	serum	real-time PCR	Dengue virus 1	96.32	206,444	1,514(0.73)
454 GS Junior	In-house-2	stool	N/A	Torque teno mini virus 5	98.89	290,647	1,226(0.42)
			N/A	Pepper mild mottle virus	98.52	290,647	1,758(0.60)
			real-time PCR	Human parechovirus 3	97.80	290,647	595(0.20)
			real-time PCR	Norovirus GII	96.28	290,647	372(0.13)
			real-time PCR	Human echovirus 9	84.83	290,647	128(0.04)
Hiseq 2000	In-house-3	swab	PCR	RSV	98.41	5,147,814	139,970(2.72)
			Nest PCR	Human coronavirus 229E	17.00	5,147,814	262(0.01)

Abbreviations: HIV, Human Immunodeficiency Virus; BoCV, Bovine enteric calicivirus; BVDV, Bovine viral diarrhea virus; RSV, respiratory syncyctial virus; PCR, polymerase chain reaction; N/A, not available.
