# Supplementary material for: VIP: an integrated pipeline for metagenomics of virus identification and discovery
Source: Sci Rep. 2016 Mar 30;6:23774. doi: 10.1038/srep23774 (PMC4824449; doi:10.1038/srep23774)
Supplement: Supplementary Information [file srep23774-s1.pdf]

VIP: an integrated pipeline for metagenomics of virus identification and discovery

Yang Li[1]†, Hao Wang[2]†, Kai Nie[1], Chen Zhang[1], Yi Zhang[1], Ji Wang[1], Peihua Niu[1] and Xuejun Ma[1]*

1. Key Laboratory of Medical Virology, Ministry of Health; National Institute for Viral Disease Control and Prevention, Chinese Center for Disease Control and Prevention, Beijing, 102206, China
2. Department of Infectious Diseases, Institute of Biomedicine, Sahlgrenska Academy, University of Gothenburg, Gothenburg, 41345, Sweden

* To whom correspondence should be addressed. Tel: +861058900810; Fax: +861058900810; E-mail addresses: maxj@ivdc.chinacdc.cn

†The authors wish it to be known that, in their opinion, the first two authors should be regarded as joint First Authors

Supplementary Methods

Range of parameters used for evaluation of classification method

Different key parameters for Bowtie2 and RAPSearch2 were computationally testing via in-silicon datasets with variable mutation rate to generate the receiver operating characteristic (ROC) curves in Figure 2. Totally there were 432 combinations for Bowtie2[1] and 6 for RAPSearch2[2].

For Bowtie2, the length of the seed substrings (L) was ranging from L7, L11, L15, L19, L23, L27, the internal function for substrings seed was ranging from i"L,1,0", i"L,2,0", i"L,4,0" and i"L,8,0", times of seed extension attempts (D) were ranging from D5, D15, D25, maximum times of "re-seed" were ranging from R0, R1, R2 and the number of mismatches in a seed alignment were ranging from N0, N1.

The expectation value or expect value (E-value) represents the probability that a given alignment to a reference in the database was due purely to chance[3]. The lower the E-value, the more significant the alignment was. For RAPSearch2, the following cutoffs of E-value were selected for the ROC curves: 10^{1}, 10^{0}, 10^{-1}, 10^{-3}, 10^{-5}, and 10^{-8}.

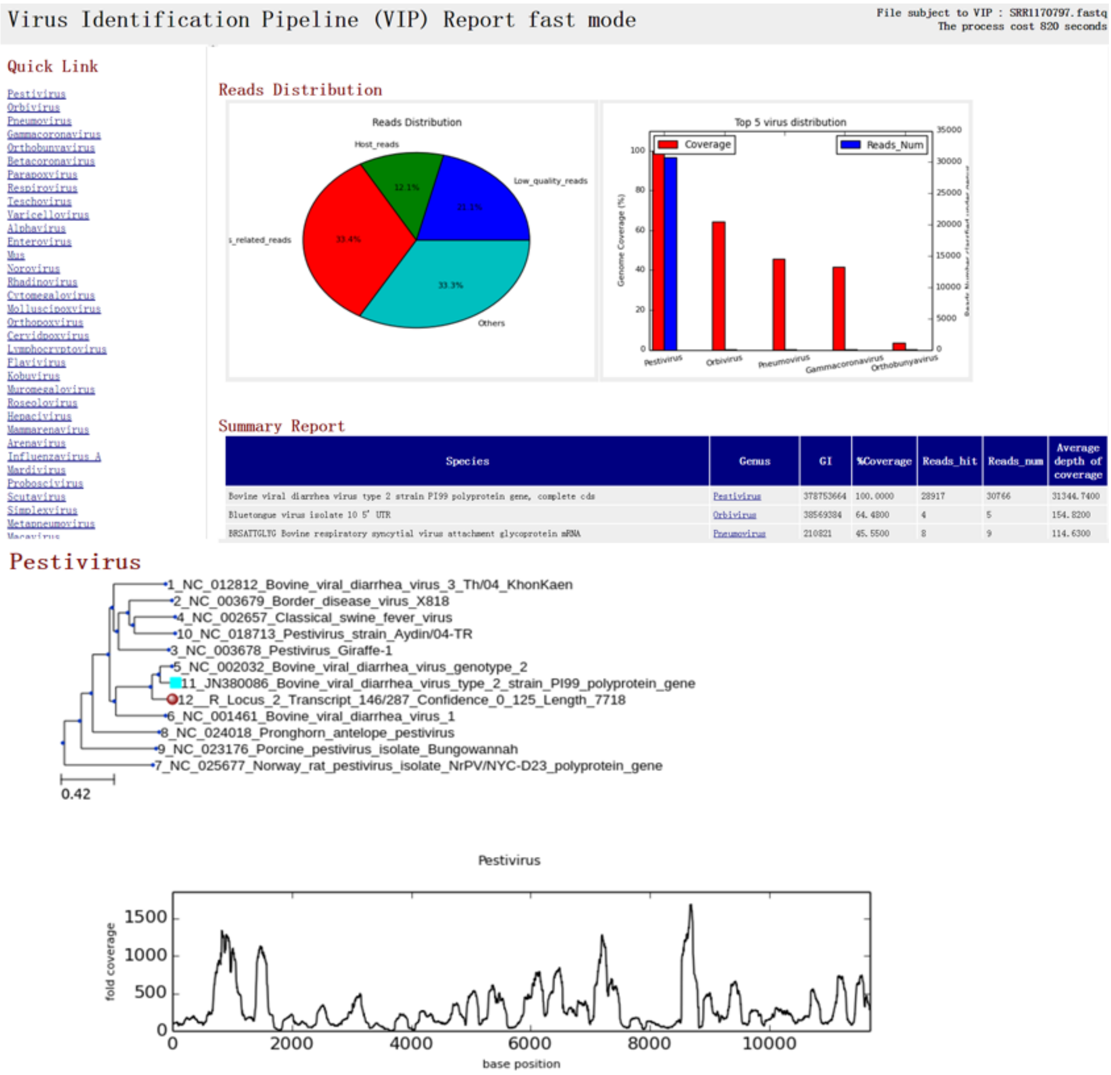

Supplementary Figure S1. Exhibition of results automatically generated by VIP using SRA1170797. Final output of VIP can be accessed from a web browser (e.g., IE, Chrome, Firefox etc.) The report included reads distribution, summary table for taxonomy classification of candidate viruses, genome coverage map and phylogenetic tree figure.

Supplementary Table S1. Sensitivity and specificity of viral detection using Virus Identification Pipeline (VIP).

SRA	Virus	Reference Accession number	Sensitivity	Specificity
**SRR1106548**	HIV	AF063223	99.83%	99.98%
**SRR1106553**	H1N1	JF915184 - JF915191	99.83%	100.00%
**SRR1170797**	BVDV	JN380086	97.03%	99.89%

Abbreviations: HIV, Human Immunodeficiency Virus; BVDV, Bovine viral diarrhea virus;

Supplementary Table S2. Summary table for siRNA NGS datasets[4] generated from Virus Identification Pipeline (VIP) in sense mode. siRNA NGS datasets were used for re-analysis by VIP. The percentages of coverage shown are with respect to the closest viral genome in the reference database.

Species	Genus	GI	%Coverage	Reads_hit	Reads_num
**Sweetpotato symptomless mastrevirus 1 movement protein (V1) gene, complete cds; and coat protein (V2) gene, partial cds***	Mastrevirus	224566911	100.00	8456	9469
**Sweet potato feathery mottle virus isolate Piu3, complete genome***	Potyvirus	223886854	100.00	224890	225259
**Sweetpotato badnavirus B, complete genome***	Badnavirus	237846577	99.14	62303	82280
**Sweetpotato badnavirus A, complete genome***	Badnavirus	336125399	88.85	23647	82280
**Sweet potato chlorotic stunt virus isolate m2-47 segment RNA1, complete sequence&**	Crinivirus	327506435	92.09	6165	9036

Abbreviations: GI: gene identifier of reference. Reads_hit: The total number of reads were mapping to the reference genome. Reads_num: The total number of reads were classified at the genus level.

*Literature report [4]

&Shown in VIP reports

Supplementary Table S3. Running time of VIP (0.1.0) versus SURPI (1.0.22) on a local server. Both VIP and SURPI were tested in default mode. Limitations of VIP include the fact that pathogens other than viruses can not be identified while SURPI can provide comprehensive identification of pathogen including parasite, bacteria. Both provided the identical candidate viral pathogens.

Dataset	Number of Reads	VIP Sense Mode (default)		SURPI Comprehensive Mode (default)	
		**Time (Seconds)**	**Pathogen**	**Time (Seconds)**	**Pathogen**
**SRR1106121**	447,073	802	Null	6210	*P. falciparum*
**SRR1106126**	1,338,420	2,735	Null	14,578	*Haemophilus influenzae*
**SRR1106548**	3,979,759	4,321	GBV-C	26,946	GBV-C
			HIV		HIV
**In-house-3 (Swab)**	5,147,814	5,185	RSV	27,573	RSV
			HcoV-229E		HCOV-229E
**In-house-4 (Swab)**	7,053,768	6,788	hPIVs	30,246	hPIVs
			HcoV-HKU1		HcoV-HKU1
			HcoV-229E		HcoV-229E
			RSV		RSV

*Hardwares: E5-2690v3*2, 128G RAM

Abbreviations: hPIVs, HIV, Human Immunodeficiency Virus; RSV, respiratory syncyctial virus; HcoV, Human coronavirus; GBV-C, GB virus C; *P. falciparum, Plasmodium falciparum.*

Supplementary Table S4. General comparisons between VIP and SURPI.

	VIP	SURPI
**General Strategy**	Subtraction to Identification	
**Alignment**	Combination of nucleotide and remote amino acid homology	
**Applications**	Virus	Bacteria, Virus, Fungi, Paratise
**Platform Supported**	454/Iontor/illumina	illumina
**Input format**	BAM/SAM/fastq/fasta	fastq
**Assemble**	Classification + Velvet + Oases (multiple k-mer)	Abyss + Minimo (fixed k-mer)
**Phylogenetic analysis**	Mafft + ETE	NULL
**Coverage Map**	Best Reference + Local Alignment	All references + Local Alignment

SUPPLEMENTAL REFERENCES

1 Langmead, B. & Salzberg, S. L. Fast gapped-read alignment with Bowtie 2. *Nature methods* **9**, 357-359 (2012).

2 Zhao, Y., Tang, H. & Ye, Y. RAPSearch2: a fast and memory-efficient protein similarity search tool for next-generation sequencing data. *Bioinformatics* **28**, 125-126 (2012).

3 Altschul, S. F. *et al.* Gapped BLAST and PSI-BLAST: a new generation of protein database search programs. *Nucleic acids research* **25**, 3389-3402 (1997).

4 Kreuze, J. F. *et al.* Complete viral genome sequence and discovery of novel viruses by deep sequencing of small RNAs: a generic method for diagnosis, discovery and sequencing of viruses. *Virology* **388**, 1-7, doi:10.1016/j.virol.2009.03.024 (2009).
